# INTERGENERATIONAL CONTACT AND THE BENEFITS FOR OLDER ADULTS

**DOI:** 10.1093/geroni/igad104.0248

**Published:** 2023-12-21

**Authors:** Xin Zhang, Wanyu Xi, David Weiss

## Abstract

Intergenerational contact can be in a variety of forms, from day-to-day interaction between family members, such as filial piety expressed by the younger generation, to artificial contact in an on-line game context. No matter what form of intergenerational contact, it is believed that such contact should be beneficial for older adults for many reasons. For example, it can help to reduce feelings of loneliness, stress and provide a greater sense of purpose. It can also help to foster a greater appreciation for differences between generations and create stronger social connections with those of different ages. It can also boost mental health, physical health, and even reduce the risk of depression. In a group of 5 individual presentations, the authors showcased that intergenerational contact could promote life satisfaction in a 5-year-interval (Ayalon & Dikla), reduce ageist attitudes toward older adults in an on-line game context (Xi, Chang, Zhang, & Ayalon), foster positive self-perception of aging (Chang & Zhang; and Tse), as well as enhance health information-seeking behaviors (Zhao, Song, & Song). These results indicated that even from different cultural background (e.g., Chinese or Americans), a positive intergenerational contact, such as providing social support to aged parents, collaborating with partners from different age groups, and even offer kindness actions toward people of a different age group could indeed be beneficial. These studies provided theoretical insights for the intergenerational contact literature, and highlighted important practical implications for potential interventions to facilitate successful aging for older adults.

